# Experimental and Numerical Analysis of the Depth of the Strengthened Layer on Shafts Resulting from Roller Burnishing with Roller Braking Moment

**DOI:** 10.3390/ma14195844

**Published:** 2021-10-06

**Authors:** Marek Kowalik, Tomasz Trzepieciński, Leon Kukiełka, Piotr Paszta, Paweł Maciąg, Stanisław Legutko

**Affiliations:** 1 Faculty Mechanical Engineering, Kazimierz Pulaski University of Technology and Humanities in Radom, 54 Stasieckiego Street, 26-600 Radom, Poland; p.maciag@uthrad.pl; 2 Faculty of Mechanical Engineering and Aeronautics, Rzeszow University of Technology, Al. Powst. Warszawy 8, 35-959 Rzeszów, Poland; tomtrz@prz.edu.pl; 3 Department of Mechanical Engineering, Koszalin University of Technology, 15-17 Racławicka Street, 75-620 Koszalin, Poland; leon.kukielka@tu.koszalin.pl; 4 Faculty of Mechanical Engineering and Computer Science, Czestochowa University of Technology, 21 Armii Krajowej Avenue, 42-201 Częstochowa, Poland; paszta@itm.pcz.pl; 5 Faculty of Mechanical Engineering, Poznan University of Technology, 3 Piotrowo Street, 60-965 Poznan, Poland; stanislaw.legutko@put.poznan.pl

**Keywords:** burnishing, hardness, material properties, roller burnishing, steel shafts, work hardening

## Abstract

The article presents the results of investigations into the depth of the plastically deformed surface layer in the roller burnishing process. The investigation was carried out in order to obtain information on the dependence relationship between the depth of plastic deformation, the pressure on the roller and the braking torque. The research was carried out according to the original method developed by the authors, in which the depth of plastic deformation is increased by applying a braking torque to the burnishing roller. In this method, it is possible to significantly increase (up to 20%) the depth of plastic deformation of the surface layer. The tests were carried out on a specially designed device on which the braking torque can be set and the force of the rolling resistance of the roller during burnishing can be measured. The tests were carried out on specimens made of C45 heat-treatable carbon steel. The dependence of the depth of the plastically deformed surface layer was determined for a given pressure force and variable braking moments. The depth of the plastically deformed layer was measured on the deformed end face of the ring-shaped samples. The microhardness in the sample cross-section and the evolution of the microstructure were both analysed.

## 1. Introduction

In many cold working processes plastic deformation occurs in the surface layer of the workpiece. The plastically deformed surface layer, with a depth between 0.1 mm and 2.0 mm [1] in steel specimens, occurs in such technologies as burnishing, thread rolling and plastic working of splines. The plastically deformed surface layer can also be observed after such technologies as turning and milling. After these machining techniques, the thickness of the plastically deformed layer is much smaller and ranges between 0.01 mm and 0.2 mm [2]. This is an advantageous phenomenon because the plastically deformed material has higher static and fatigue strength. The problem of determining the depth of a plastically deformed layer is difficult, both theoretically and experimentally. In industrial practice, burnishing is used in two main types of plastic working: slide burnishing (SB) and roller burnishing (RB). The purpose of slide burnishing is to obtain a low surface roughness of the workpiece after turning or milling. The process takes place with low pressure forces [3] using a stationary tool that is hardened steel or a ceramic ball [4]. Plastic deformation of the surface layer only takes place in the area of the peaks of the surface roughness [5]. In rolling burnishing, the tool is a rotary ball or a roller with a toric profile [6], which is pressed by an adjustable pressure.

The plastic deformation created by the roller is a displacement of the material that flows from the peaks into the valleys under pressure, and results in a surface finish with a strain-hardened surface [7,8]. The purpose of RB is plastic deformation of the surface layer in the area of the peaks of the surface asperities in order to obtain a roughness of Ra = 0.08–0.15 µm [9]. The roller burnishing process is used to achieve a high-quality surface finish strengthened through the work hardening phenomenon [10]. Kowalik [11] has shown an increase in yield strength of up to 20% in the plastically deformed layer, depending on the burnishing conditions.

Hertz [12] drew on elastic theory to examine the relationships between pressure *p* and compression load *F*. The state of triaxial compression occurring in the surface layer may cause contact stresses to exceed the value of the yield point. Bielajew [13] observed the change of stresses in the surface layer under the influence of loading and concluded that the greatest deformations of the material occur at a certain depth beneath the surface layer, at the so-called Bielajew points. Bielajew’s theory was used to determine the equivalent stresses in von Mises theory [14].

**Figure 1:**
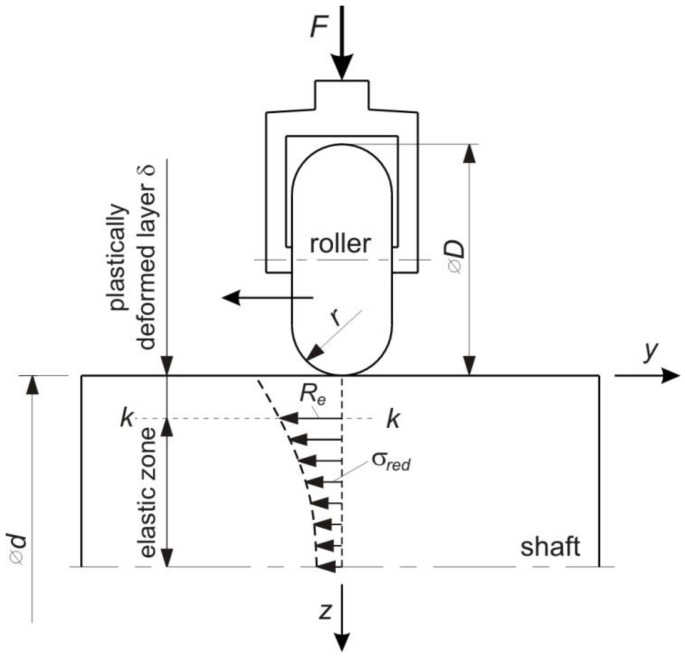
The roller burnishing scheme used to calculate the thickness of the plastically deformed surface layer along the axis of action of the thrust force (at *x* = *y* = 0): *D*—roller diameter, *r*—roller radius, *d*—shaft diameter, *δ*—depth of plastic deformation, *R_e_*—yield stress.

Kowalik et al. [15] proposed a theoretical method of determining the depth of plastic deformation in the RB, based on the Hertz theory. The solution to the problem proposed by Jezierski and Mazur [16] was based on the simplifying assumption that there are zones of plastic deformation and elastic deformation in the surface layer. Figure 1 shows a diagram of this method and the quantities used for the calculations.

The distribution of elastic stresses during burnishing is presented as a dashed line. The stress value at depth *δ* is equal to the yield stress. Below this value, the stress character is elastic [17]. The character of the stress distribution in the plastic deformation zone does not change substantially from the elastic stress distribution. The depth value *δ* separating these two zones can be determined on the basis of the Hertz-Bielajew strain theory, which determines the geometrical relationships between two plastically deformed bodies. The contact of the roller with the shaft surface represents a symmetrical case in which the contact surface has an ellipsoidal shape.

After complicated calculations based on differential geometry, both Jezierski and Mazur [16] and Kowalik [17] obtained a solution to the problem of the depth of the deformed layer *δ*, depending on the roller pressure force *F*, material parameters (*Re*—yield stress of the workpiece material, *ν*—Poisson’s ratio and *E*—Young’s modulus) and the contact geometry of the roller and the shaft (*d*—shaft diameter, *D*—roller diameter, *r*—roller radius). For a specific material described by the *Re*, *ν*, *E* parameters, the roller pressure force *F* had the greatest impact on the depth of the plastically deformed layer. The contact geometry of the shaft and roller was less significant. Due to the complexity of the general solution, its practical use requires the application of numerical methods, nomograms or tabulated values. Due to this fact, the solution is complicated and of hardly any benefit for industrial use. Calculations of the depth of the plastically deformed layer conducted by Teimouri and Amini [18] were based on the general solution of the contact problem developed by Hertz [12]. They presented a model of the depth of the plastic deformation of the surface layer for static and dynamic deformation in ultrasonic ball burnishing and verified it with a finite element method (FEM) simulation and hardness measurements. The model takes account of the effect of factors such as static force, feed rate, ball diameter and ball material, as well as ultrasonic vibration parameters such as amplitude and frequency. To incorporate the effect of feed rate, two important parameters, linear coverage coefficient and overlapping ratio, have been defined and have contributed to the model. Assessment of the depth of the plastically deformed layer is particularly important for the determination of the fatigue strength.

The depth of the hardened layer is closely correlated with the value of the fatigue strength. It was found that the ratio of the depth *δ* of the hardened layer to the shaft diameter *d* [16] is useful in determining this depth. In recent investigations, this ratio was found to be *δ*/*d* ≥ 0.05 [16]. The depth of the hardened layer should be as deep as possible, but with the simultaneous condition that no cracks should occur on the roller surface during burnishing [19,20,21].

In recent decades, research has primarily focused on the process of slide burnishing and roller burnishing of various materials. Researchers determined the correlation between pressure forces, feed rate, surface microhardness, burnishing speed, and roughness, before and after burnishing. Balland et al. [22] investigated the influence of burnishing parameters on material properties. Finite element-based computations showed that the mechanism of creating ridges plays an important role in providing the required surface quality. Burnishing depth and feed rate play a key influence on the microhardness of the material and the strength parameters of the burnished material [23]. Loh et al. [24] and Hassan and Maqableh [25] found that the use of a different lubricant and the initial surface roughness and hardness of the workpiece have significant effects on the burnishing process. The results of investigations by Franzen et al. [26] have shown that the process parameters of the roller burnishing process have a strong influence on the tribological properties of the friction elements and their surface topology. The surface asperities of the coating are flattened by the roller burnishing process and the contact area between the finished surface and the sheet is increased. Okada et al. [27] found that burnishing force, feed rate, roller-inclination angle, and number of tool passes had the greatest impact on the integrity of the burnished surface in roller burnishing with an active rotary tool. Experimental investigations conducted by Kowalik and Trzepiecinski [28] have shown that the depth of plastic deformation of the surface layer in the burnishing process for strengthening C45 shafts depends on the state of stress in the workpiece material.

The utilitarian goal of the research presented here is to develop a very efficient and cheap method of formation of the surface with both high surface smoothness and shape accuracy. Slide burnishing and roller burnishing technologies are relatively well researched, however many problems have appeared with the hardening burnishing (HB) technology, which have to be resolved. The main issue is how to set the basic process parameters such as roller pressure *F*, roller feed *F* and rolling speed *v*. Another problem is what the roll geometry (roller diameter *D* and rounding radius of roller *r*) should be in relation to the roller diameter *d* in order for the optimum depth of plastic deformation *δ* and high surface smoothness without cracks and pitting to be obtained.

Kowalik et al. [28,29] developed an original method for determining the depth of deformation of the plastically deformed layer during roller burnishing, based on the observation of the front surfaces of ring specimens. The method thus developed involves increasing the depth *δ* of plastic deformation in the surface layer by applying a braking moment on the burnishing roller, while maintaining the rolling nature of the burnishing process. In a recent paper by Kowalik et al. [15], an analytical method was developed for determining the depth of the plastically deformed layer on the basis of the Hertz–Belyaev theory. The depth of deformation was obtained as a function of the process parameters: material strength, burnishing force and roller radius. Analysis of the RB process, in which a braking moment was applied to the roller, was first analysed by the authors in papers [28,29]. However, the results only applied to the RB process with braking moment. Moreover, only the roller pressure force *F* = 3 kN was analysed. This article compares the results of the microhardness distribution and the depth of the surface layer for two variants of the RB process realised with and without braking the roller. The rolling resistance force values were also determined for a wide range of burnishing force changes between 1 kN and 5 kN. The experimental results are supplemented by the results of numerical modelling of both variants of RB analysed. Using a specially built test stand, the change in the rolling resistance of the roller during the RB process was also presented and discussed. The research demonstrated that the braking force applied to the roller causes a change in the stress state in the burnishing zone and an increase in the depth of the plastically deformed layer.

## 2. Materials and Methods

### 2.1. Material

The first series of tests were carried out on samples made of C45 carbon steel subjected to annealing. Selected mechanical parameters of theC45 steel are presented in Table 1. Chemical composition of the steel used in the tests is listed in Table 2.

### 2.2. Methods

#### 2.2.1. Background

**Figure 2:**
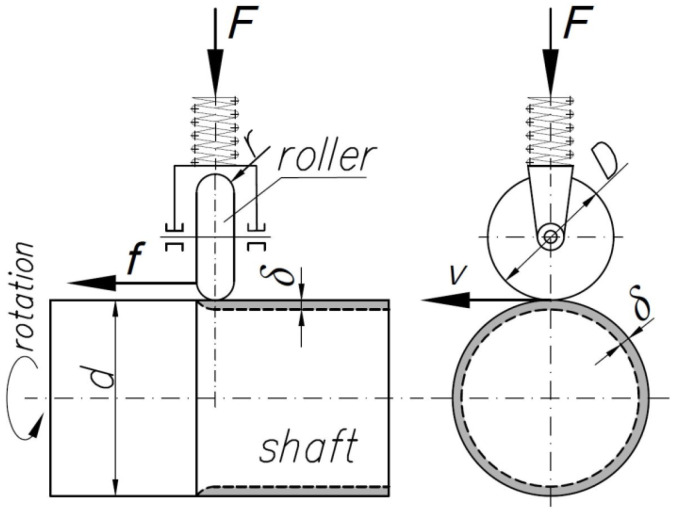
Schematic diagram and parameters of conventional roller burnishing.

Classic roller burnishing is carried out according to the scheme shown in Figure 2. The aim of this process is to obtain a large depth of the plastically deformed surface layer *δ* and appropriate surface roughness.

The cold-deformed material in the outer layer has greater strength and hardness due to the work hardening phenomenon, as well as greater fatigue strength.

The theoretical analyses [15,17] and experimental studies [10] showed that the roller pressure force has the greatest impact on the depth of the plastically deformed surface layer. The contact geometry described by the roller and the shaft diameters and roller radius are much less significant. Experimental studies have shown that with adequately established technological parameters (*F*, *D*, *r*, *d*, *f*, *v*), the depth of plastic deformation *δ* reaches the maximum value.

Hardening burnishing technology is carried out using a spring to exert pressure on the workpiece (Figure 2). The burnishing force does not depend on the size of the burnishing allowance but depends on the settings of the spring elements of the tool, such as the pneumatic and hydraulic actuators. The resulting parameter of the RB process is the depth of the plastically deformed layer *δ*. Inappropriate selection of the technological parameters, mainly burnishing forces, may lead to the destruction of the workpiece through peeling, surface cracks, etc. [31].

**Figure 3:**
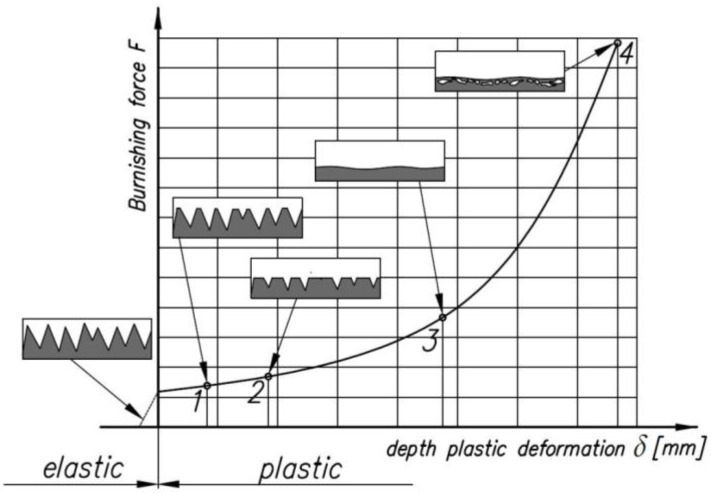
The effect of burnishing force on the deformation of surface asperities and the depth of the plas-tically deformed layer: 1—flattening the roughness asperities, 2—severe flattening of the surface topography, 3—completely flattened surface, 4—work hardened subsurface zone.

A diagram of the effect of the burnishing force on the deformation of the surface asperities and the depth of the plastically deformed layer is shown in Figure 3. Plastic deformation occurs at the peaks of the surface asperities (between points 1 and 2 in Figure 3) with a minimal increase in the value of the burnishing force *F*. Plastic deformation then occurs on the entire area of the surface asperities (between points 2 and 3), and the workpiece surface is smoothed at point 3. Further increase of the pressure (between points 3 and 4) causes an increase in the depth of the plastically deformed layer until cracks and pitting occur, which show that the surface has been destroyed (point 4).

#### 2.2.2. Roller Burnishing

The purpose of burnishing was to obtain the maximum possible depth of plastically deformed surface layer with constant burnishing parameters. The burnishing samples (rings) were attached to a special mandrel. During burnishing, the rings could not be pulled apart axially. The roller pressure *F* was changed within the range 1–5 kN. The dimensions of the samples, the roll geometry, and the burnishing parameters are listed in Table 3.

**Figure 4:**
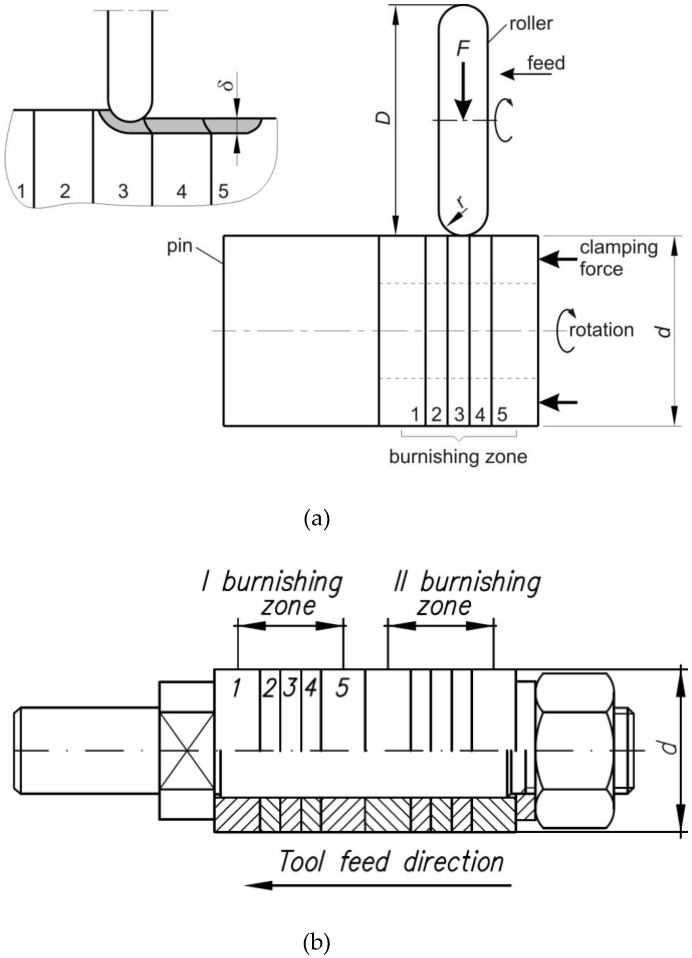
Schematic diagram of (**a**) the test method and (**b**) mandrel with mounted rings: 1, 5—stopper rings; 2–4—operating rings.

Figure 4a shows a schematic diagram of the test method. The design of the mandrel with a package of six rings is shown in Figure 4b. The rings (1 to 5) were sat tightly on the mandrel, and they were compressed by means of a nut. The tightening torque is selected so that the axial force compressing the rings is at least 30 kN.

**Figure 5:**
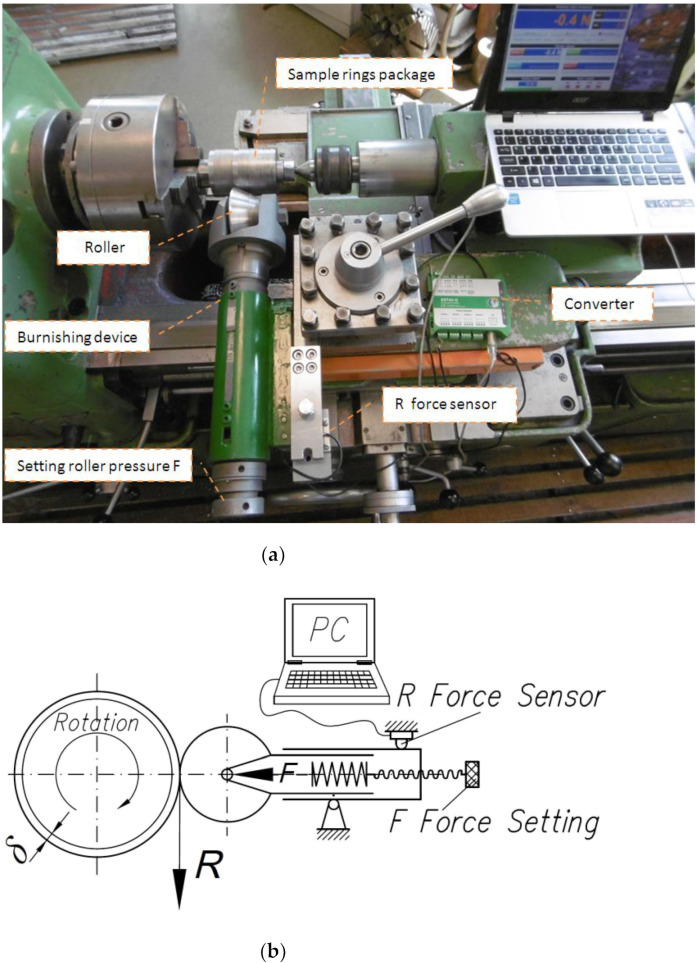
(**a**) Photograph and (**b**) schematic diagram of the stand used for testing the roller burnishing process.

Roller burnishing was carried out on a lathe with a device in which the roller pressure force *F* is adjustable through a spring (Figure 5a,b). The device is equipped with a sensor that allows the rolling resistance force *R* of the roller to be measured.

The specimens for microstructural analysis were hot-embedded into conductive Bakelite resin with carbon filler. After embedding, the specimens were prepared by machine grinding and polishing. Microstructure was revealed by etching in 2% Nital (98 mL ethanol + 2 mL HNO_3_). The microstructure was examined by a Nikon Eclipse MA200 optical microscope (Nikon Corporation, Tokyo, Japan) coupled with a Nikon DS-Fi2 digital camera.

#### 2.2.3. Roller Burnishing with Braking Torque

**Figure 6:**
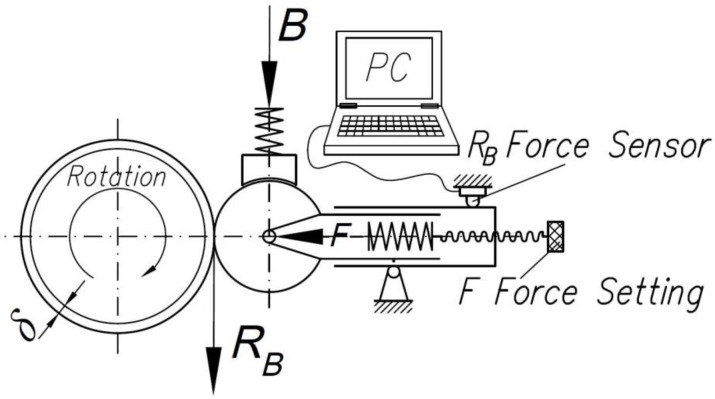
Schematic diagram of roller burnishing with braking torque applied directly to the roller.

**Figure 7:**
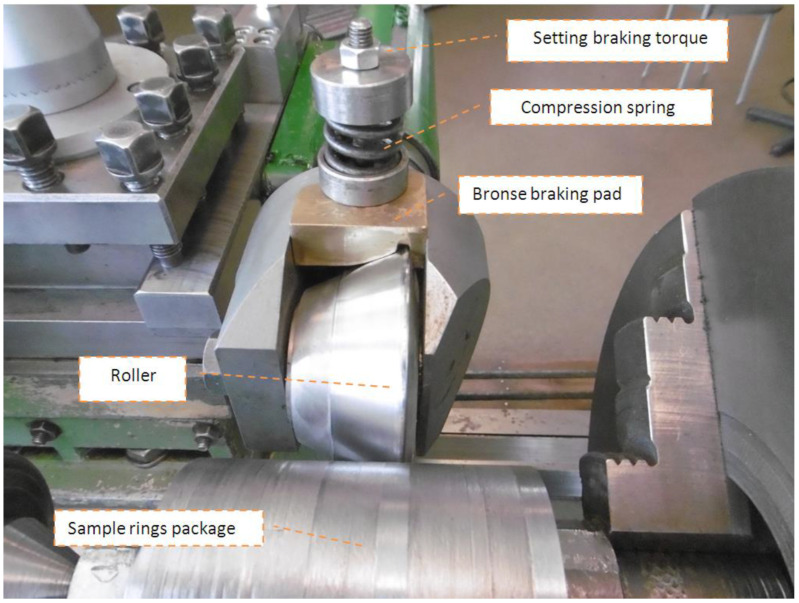
Burnishing device with mounted roller brake.

The second series of investigations was conducted using the new method developed by the authors. The method involves the application of a braking torque to the burnishing roller, thanks to which burnishing takes place in a triaxial asymmetric state of stress. A schematic diagram of the method is presented in Figure 6. The tests were carried out on the test stand shown in Figure 5a, which was equipped with a brake acting directly on the burnishing roller (Figure 7).

### 2.3. Numerical Modeling

**Figure 8:**
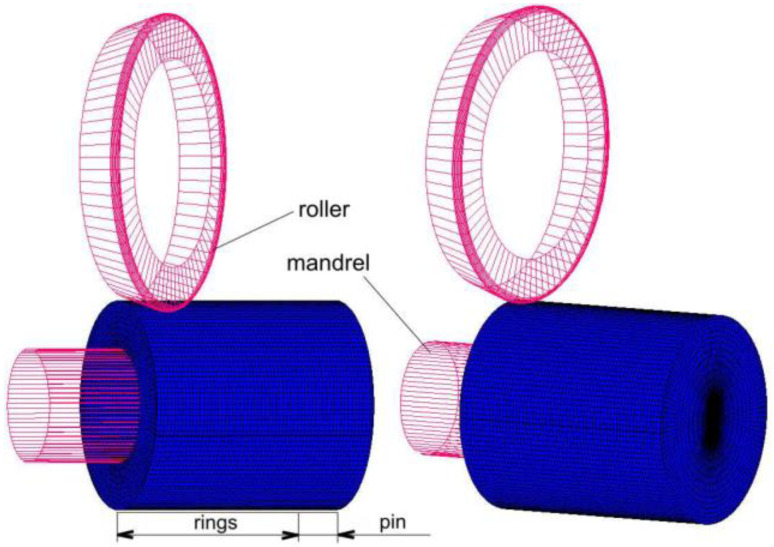
Numerical model of roller burnishing.

A numerical model of the roller burnishing process with roller braking was built in the MSC Marc program for modelling physical phenomena using the finite element method. The geometrical model of the shaft, mandrel and roller corresponded to the real geometrical dimensions (Figure 8). In order to limit the size of the numerical task, the calculations were performed for a model containing five rings. The ring and pin models included 72,000 brick-type finite elements. The outer surface of the shaft, where large deformations are expected, has been subdivided into smaller elements. The mesh size was determined on the basis of a sensitivity analysis. Simulations were carried out with different sizes of finite elements on the surface of the rings in the range from 0.5 to 2 mm. The deformation values at the assumed point in the model coincided with a decrease of the mesh element. The reduction of the mesh size from 1 to 0.5 resulted in a 3.4% reduction in the deformation value. However, the computation time increased by 450%. Thus, for further calculations, a model was adopted that had elements with an edge size of about 1 mm on the surface of the rings.

The mandrel and roller models have been modelled as non-deformable surfaces. A pressing force of 3 kN was applied to the roll. The mechanical parameters of the shaft material were assumed according to Table 1. It was assumed that the elastic-plastic material model with non-linear work hardening applied. For the burnishing without braking moment, the value of the friction coefficient in the friction model, which depends on the workpiece-tool slip velocity, was assumed to be *μ* = 0.1 [10]. In the case of burnishing with braking applied to the roller, the coefficient of friction has been estimated as the proportion between the rolling resistance force and slide burnishing force. The value of the friction coefficient has been determined experimentally as a ratio of rolling resistance force (tangential force) and burnishing force (normal force).

## 3. Results and Discussion

### 3.1. Experimental Investigation

Determining the depth of the plastically deformed surface layer is difficult because the zone of elastic and plastic deformation does not have a clear boundary. Most researchers determine the depth of the plastically deformed layer on the basis of the hardness distribution in the cross-section. This is not a precise method. Wang et al. [32] and Sonmez et al. [33] found a significant increase in the material strength and small changes in the hardness as a function of deformation in cold forming. Agrawal and Singh [34] obtained a 5% increase in the hardness of steel samples with 60% of the material undergoing plastic deformation.

**Figure 9:**
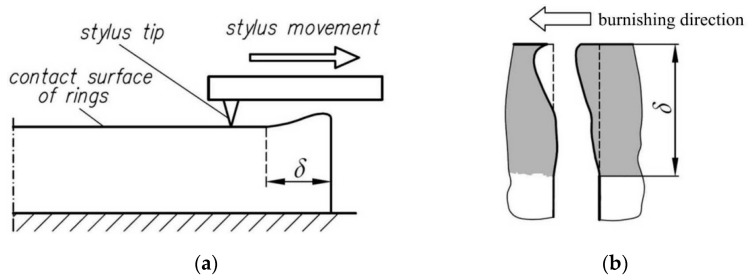
(**a**) Deformation of the side surfaces of adjacent rings corresponding with the plastic deformation depth *δ* and (**b**) measurement of the depth *δ* of the plastically deformed layer.

The method of measuring the depth of the plastically deformed layer involves determining the depth on the basis of the results of profilographometric measurements on carefully prepared (grinding) front surfaces of disconnected rings, which are joined on a mandrel and compressed by an axial force during burnishing (Figure 9). The profilographometric measurements were carried out on the front surfaces of the rings with the use of a profilometer coupled with a digital multimeter and computer.

Figure 9a enlarged the contact area in the deformed zone of adjacent rings. Figure 9b shows a schematic diagram of deformations on the surfaces of the rings after their disconnection. The measurements of the layer thickness *δ* made during burnishing of the roller with different pressure force values are listed in Table 4.

The second series of samples was burnished with the same parameters, but with braking torque applied on the roll. The braking torque was selected in such a way that the burnishing took place on the border between roller and slide burnishing. The pressure force on the roller brake was selected individually for the individual values of the pressure force *F* so that the braking torque did not stop the roller during burnishing. Burnishing was carried out with the maximum possible rolling resistance applied to the roll, while maintaining the rolling nature of the burnishing process. This means that a slight increase in braking torque would make the burnishing process stop.

**Figure 10:**
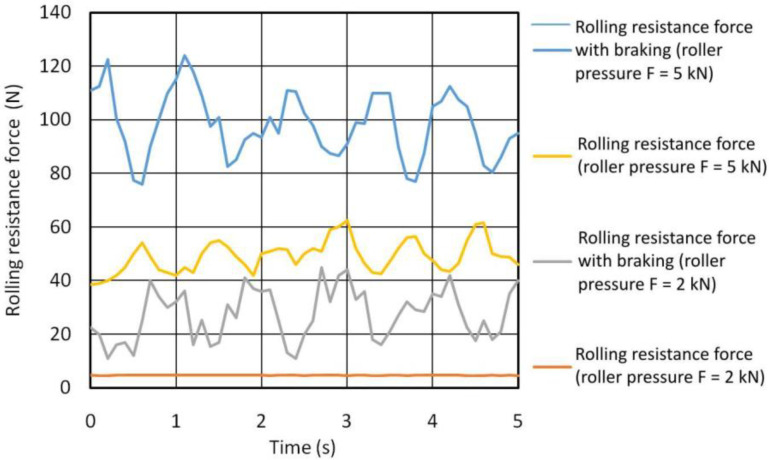
Rolling resistance force of the roller with and without braking applied to the roller.

Figure 10 summarises the rolling resistance force *R* of the roller for burnishing with the roller pressure force *F* = 5 kN and *F* = 2 kN. The roller pressure forces *F* for burnishing with the braking moment, and the rolling resistance force *R*_B_ of the roller are also shown. By analysing the values given in Table 5 and the recorded variation of the force, it can be seen that the roller resistance force *R*_B_ increases with roller pressure *F*. Therefore, the most effective application of braking on the roller in order to increase the deformation depth is at a higher roller pressure force. Relatively large fluctuations of the rolling resistance force result from the application of direct braking of the roller under dry friction conditions [35]. In the process of burnishing with the use of roller braking, a significantly greater depth of plastic deformation can be obtained.

**Figure 11:**
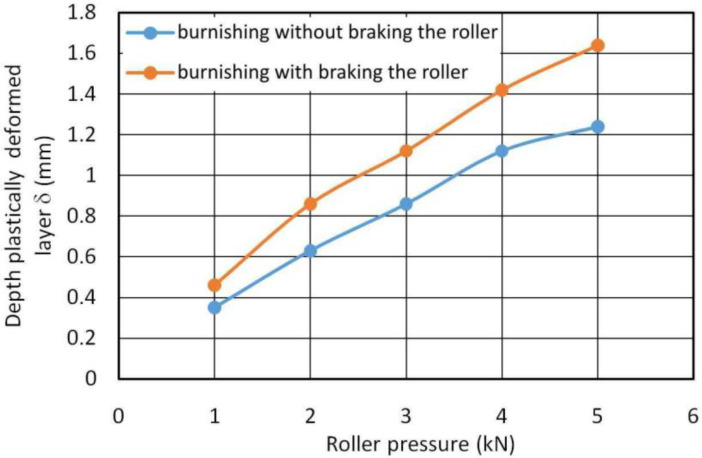
Effect of roller pressure on the depth of the plastically deformed layer for burnishing with and without braking applied to the roller.

The depth of the plastically deformed layer was determined on the basis of the profilogram of the end faces of the rings in the burnishing zone according to the method presented in Figure 9. Figure 11 shows the dependence of the depth of the plastically deformed layer as a function of the roller pressure force for burnishing with and without braking. In the process of hardening burnishing with the use of roller braking, a significantly greater depth of plastic deformation is obtained due to the asymmetrical state of stress during deformation in the zone of contact between the rings and the roller.

**Figure 12:**
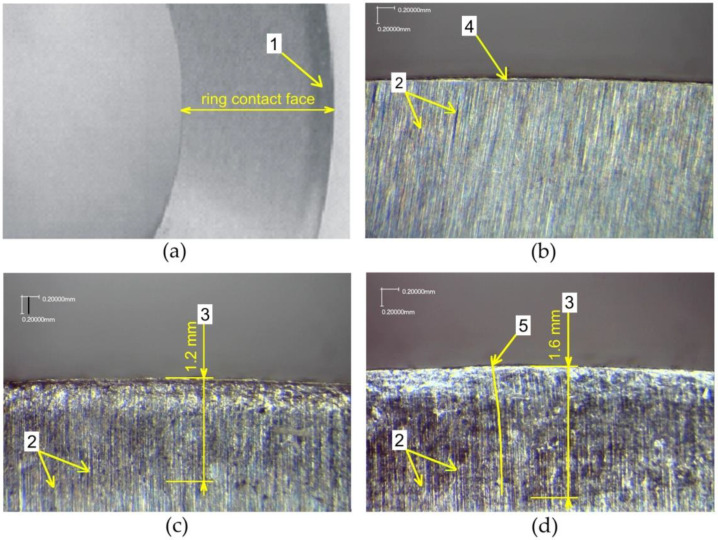
(**a**) End face of the burnished ring, (**b**) end face of the non-burnished ring, (**c**) end face of the bur-nished ring without braking torque, (**d**) end face of the burnished ring with braking torque of the roller: 1—plastically deformed zone, 2—grinding tracks, 3—depth of plastically deformed zone, 4—subsurface zone, 5—curving of grinding tracks.

During the observation of the front surface of the measuring rings (Figure 12a), it was noted that the deformation of the surface layer can be observed along the line of the grinding traces shown in Figure 12b. The marks of separation of the deformed zone and non-deformed zone are most visible when the lines of the sanding marks run in a direction perpendicular to the cylindrical surface of the ring. In the case of burnishing without braking torque, a clear fold is visible (Figure 12c). During burnishing with the application of braking torque, the transition from the non-deformed area to the plastically deformed layer is not so clear, however the bend of the grinding lines can be observed (Figure 12d).

The deformation of the surface layer can be observed on the faces of the rings under slight magnification. Observation is facilitated by the grinding marks on the faces of the rings. The characteristic zones are marked in Figure 12c,d and their dimensions are given. In the case of conventional roller burnishing, parallel grinding marks are observed both in the non-deformed zone and with plastic deformation. A different character of plastic deformation is observed for samples burnished with braking torque (Figure 12d). In the zone of transition from the non-deformed zone to the plastically deformed area, the grinding marks are bent in the direction of action of the braking torque. With the same pressure force of the burnishing roller *F*, the depth of plastic deformation *δ* is much greater if we apply braking torque.

**Figure 13:**
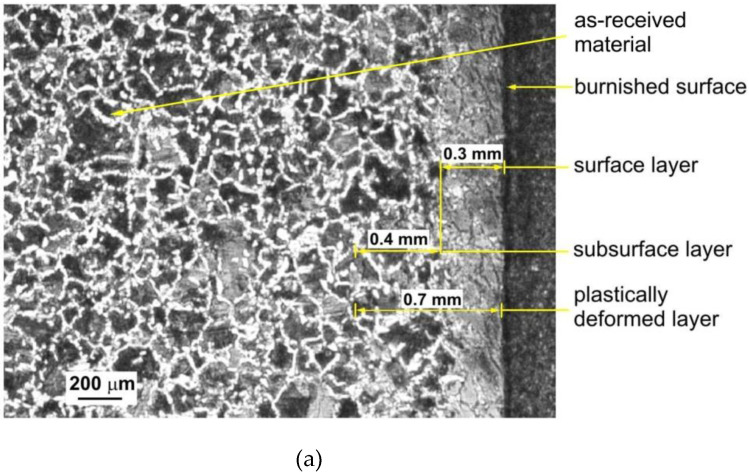

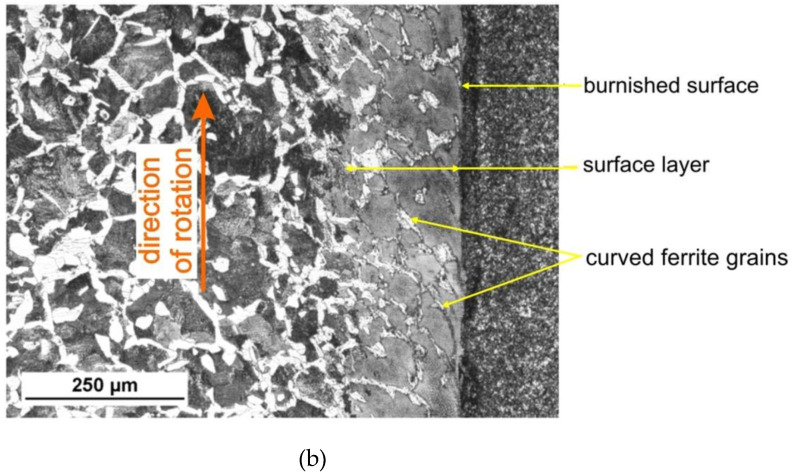
Structure of the plastically deformed surface layer of specimen burnished with braking moment: (**a**) magnification ×200, (**b**) magnification ×500.

Measurements of plastic deformation on the end faces were verified on metallographic specimens. Specimens from cross and longitudinal sections of the rings used for making the microsections were taken from the rings, polished and etched to enable observation of the microstructure. The samples shown in Figure 13 were taken from rings burnished with the roller pressure force *F* = 5 kN with braking torque. The structure of the C45 steel ring in the non-deformed zone consists of dark pearlite grains separated by white ferrite network.

A deformed layer was identified which extended approximately 0.7 mm into the surface. The plastically deformed surface layer has two characteristic zones. The near-surface zone consists of intensely deformed perlite grains and finely divided ferrite grains. The depth of this zone is about 0.3 mm and is clearly separated from the subsurface zone. The depth of the subsurface zone is marked. The border of the transition to the subsurface zone is very clear and easy to determine.

Figure 13b shows the structure of the surface layer in a cross-section under a higher magnification. The ferrite and perlite grains are highly stretched and curved in the opposite direction to the rotation of the ring. The bend of the ferrite and pearlite grains is identical to the curvature of the grinding marks on the front surface of the rings observed in Figure 12d. The depth of plastically deformed layer that can be identified from the observation of the structure is 0.7 mm (Figure 13a), while the one observed at the faces of the rings is 1.6 mm (Figure 12d). This value, more than twice as high, results from small deformations of the microstructure that are impossible to observe on a metallographic microscope. The researchers [32,33,34] encountered the same problem, who for 60% plastic deformation observed minimal changes in the microstructure and an increase in hardness.

The transition boundary between the non-deformed material and the plastically deformed layer is more difficult to determine because it is difficult to identify very small plastic deformations. The area in which there is a change in the nature of the ferrite network can be observed as the boundary between these zones. In the non-deformed zone, the ferrite network is clearer, the grains are wider and there are numerous branches and thickenings. In the zone of plastic deformation, the ferrite grains are thinner, and the branches and thickenings disappear.

Hardness measurements were made on the transverse and longitudinal sections. The load of the indenter was selected so that the imprint during the test covered several grains. Because the ferrite and perlite grains have significantly different hardness, the risk of measuring the hardness of individual grains was avoided. In this way, the average character of the hardness measurement was obtained. The measurement was made from the surface into the material with steps of 0.05 mm. The first hardness measurement was made at a distance of 0.05 mm from the surface.

**Figure 14:**
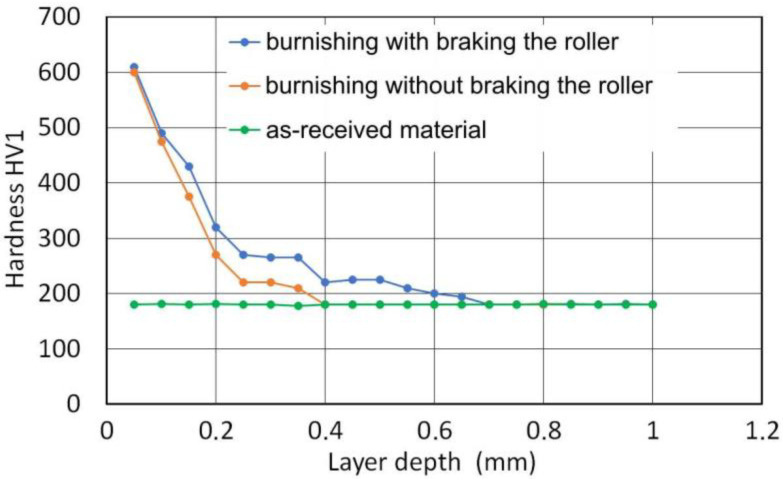
Effect of the surface layer depth on the cross-section hardness HV1 of ring (roller pressure force *F* = 5 kN).

Figure 14 shows the hardness characteristics of the surface layer. In the near-surface layer in the depth range of up to approx. 0.3 mm, we can observe clear changes in hardness which correspond to the changes in the microstructure observed on the metallographic specimens (Figure 13). In the deeper layers of the material, changes in hardness are small, despite the fact that clear changes in the microstructure can be observed. In the outer layer of the material, the hardness after burnishing increases approximately three times. Burnishing with braking torque provides greater hardness in the material layer located between 0.1 and 0.6 mm compared to the variant of burnishing without braking. This proves that the braking of the roller results in a more intense impact of the roller on the workpiece, which has a positive effect on the strain hardening of the surface layer. This is one of the ways to increase the fatigue strength of machine components.

As a result of cold plastic deformation, the material reaches a certain extreme of hardness and mechanical strength at which the processes of microcracking and pitting begin. The hardness of a material cannot be increased above a certain value for each material. Therefore, in the near-surface layer, the hardness of the material in the area of contact with the roller is similar in both RB processes analysed (Figure 14). However, there are differences in the depth of the plasticised layer (Figure 11).

### 3.2. Analysis of Stress State in RB

**Figure 15:**
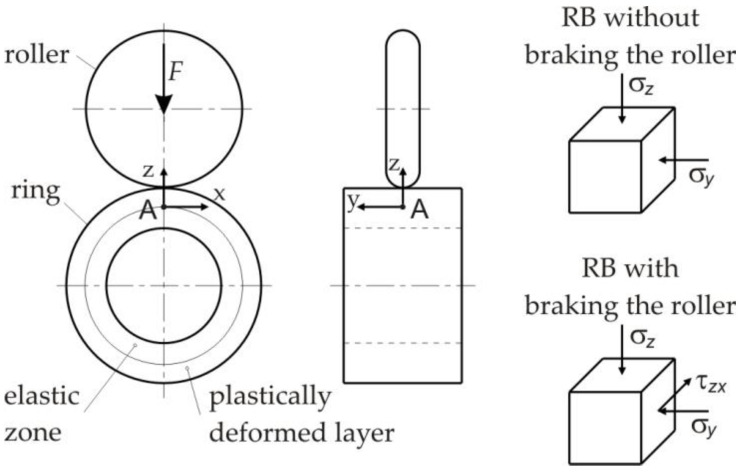
Stress tensor components for point A for variants of RB with and without braking the roller.

In the burnishing zone under the roller, two areas can be distinguished: an elastic zone and a plastically deformed layer (Figure 1). Based on this assumption in the work [25], the depth of the plastically deformed layer was calculated using the relationships from the theory of elasticity. In the layer of plasticised material, the equivalent stress *σ_eq_* according to the von Mises yield criterion corresponds to the yield stress *R_e_*. Therefore, for the points lying on the boundary of the layers subjected to elastic and plastic deformation, the relations of the theory of elasticity can be used. Figure 15 shows the components of the stress tensor for point A lying directly under the roller on the border of the elastic, and plastic zones for both variants of the RB process analysed.

The components of the stress tensor in the general form are:

(1) $$\sigma_{eq}=\left[\begin{array}{ccc} \sigma_{x} & \tau_{xy} & \tau_{xz}\\ \tau_{yx} & \sigma_{y} & \tau_{yz}\\ \tau_{zx} & \tau_{zy} & \sigma_{z} \end{array}\right]$$

where *σ_z_*, *σ_y_* and *σ_z_* are normal components, and *τ_xy_*, *τ_xz_* and *τ_yz_* are tangential components of stress tensor. It should be noted that according to the stress theory *τ_xy_* = *τ_yx_*, *τ_xz_* = *τ_zx_* and *τ_yz_* = *τ_zy_*.

After taking into account the stress tensor components characterising burnishing (Figure 9) in Equation (1), the tensors obtained for classic burnishing with braking *S_RB_* and without braking *S_R_* are as follows:

(2) $$S_{R}=\left[\begin{array}{ccc} 0 & 0 & 0\\ 0 & \sigma_{y} & 0\\ 0 & 0 & \sigma_{z} \end{array}\right]$$

(3) $$S_{RB}=\left[\begin{array}{ccc} 0 & 0 & \tau_{xz}\\ 0 & \sigma_{y} & 0\\ \tau_{zx} & 0 & \sigma_{z} \end{array}\right]$$

where: *σ_z_* is the stress component originating from the roller pressure force *F*, *σ_y_* is the stress component originating from the feed force of the roller, *τ_xz_* is the stress component originating from the roller braking force.

The stress tensor being considered at point A for burnishing with braking the roller takes into account the additional tangential component *τ_xz_* coming from the braking force of the roller.

The general dependence on the equivalent von Mises stress has the form:

(4) $$\sigma_{eq}=\sqrt{\frac{1}{2}{\left(\sigma_{x}-\sigma_{y}\right)}^{2}+{\left(\sigma_{y}-\sigma_{z}\right)}^{2}+{\left(\sigma_{z}-\sigma_{x}\right)}^{2}+3\left(\tau_{xy}^{2}+\tau_{yz}^{2}+\tau_{zx}^{2}\right)}$$

The values of the components *σ_z_, σ_y_* and *τ_zx_* are proportional to the roller pressure force *F*, the value of which has been measured experimentally. The feed force of the roller was 0.25·*F* and the braking force was about 0.03·*F*. To simplify the notation, let us denote *σ_z_* as σ. Then *σ_y_* will be equal to 0.25*σ* and *τ_zx_* = 0.03·*σ*. After introducing these values into Equation (4), we obtain the values of equivalent stress for burnishing without ($\sigma_{eq}^{R}$) and with ($\sigma_{eq}^{RB}$) braking the roller:


(5) $$\sigma_{eq}^{R}=\sqrt{\frac{1}{2}{\left(-\frac{1}{4}\sigma \right)}^{2}+{\left(-\sigma \right)}^{2}+{\left(\frac{1}{4}\sigma -\sigma \right)}^{2}}=1.15\sigma$$

(6) $$\sigma_{eq}^{RB}=\sqrt{\frac{1}{2}{\left(-\frac{1}{4}\sigma \right)}^{2}+{\left(-\sigma \right)}^{2}+{\left(\frac{1}{4}\sigma -\sigma \right)}^{2}+3\times 0.03^{2}}=1.18\sigma$$

The results show that at the same roller pressure *F* at the point A considered, the value of the equivalent stress for the RB process with braking the roller is greater than for the RB process without braking the roller. Due to the additional tangential component of the stress tensor, the depth of the plasticised layer is greater for RB with braking the roller.

### 3.3. Numerical Modelling

**Figure 16:**
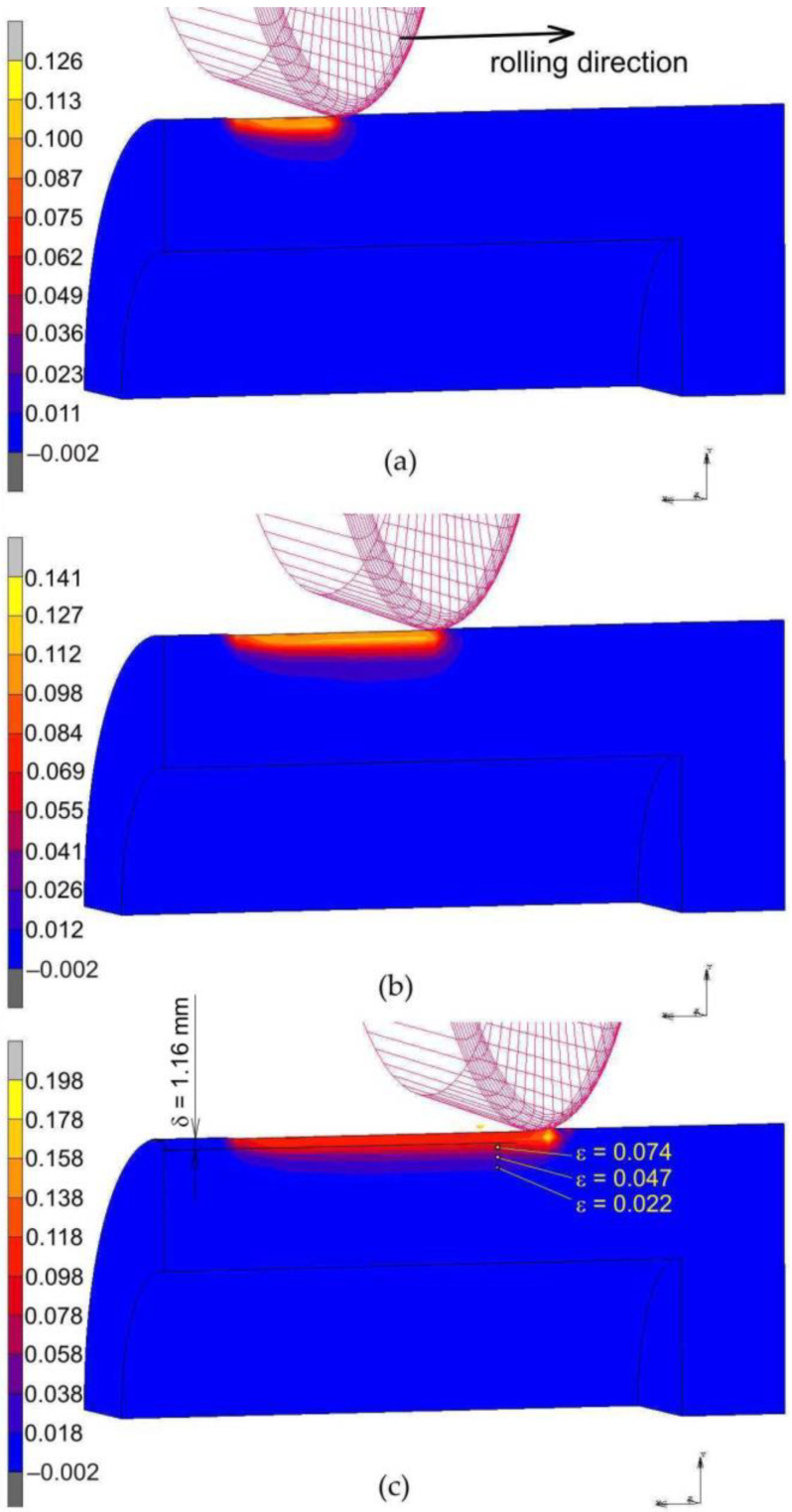
Distribution of equivalent plastic strain ε after roller burnishing with braking moment across the width of the rings packet: (**a**) 10 mm, (**b**) 20 mm and (**c**) 30 mm.

**Figure 17:**
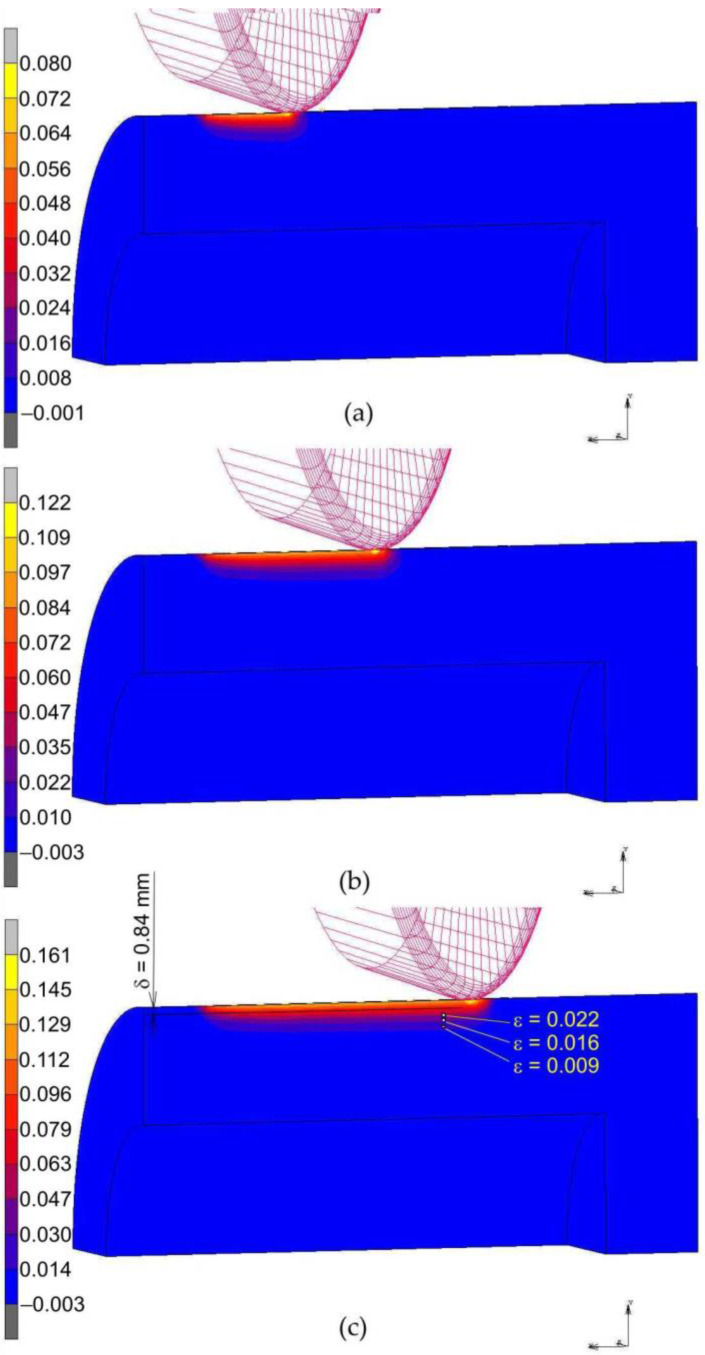
Distribution of equivalent plastic strain ε after roller burnishing without braking moment across the width of the rings packet: (**a**) 10 mm, (**b**) 20 mm and (**c**) 30 mm.

Figure 16 and Figure 17 shows the distribution of equivalent strains on the cross-section of the rings. For the sake of clarity in the presentation of the results, the cross-section was limited to ¼ of the model. During the burnishing process, the depth of the plastically deformed layer is very even, and the most plasticised zone is located in the subsurface layer of the material, directly impacted under the roller. The depth of the plasticised layer determined by the finite element method is about two-times greater than that estimated in the experimental tests of RB without braking the roller (*δ* = 1.16 mm). In the case of the RB process with braking the roller, the depth of the plastically deformed layer, based on the numerical simulation, is approximately 2.85 mm. Meanwhile, the depth of the plasticised layer determined experimentally based on the analysis of the structure of the ring faces is *δ* = 1.6 mm. However, it should be remembered that the experimental estimate of the depth of the plastically deformed layer is burdened with a large error resulting from the inability to detect the area of small plastic deformations in the material. The numerical model did not take into account the local grain orientation and the associated favourable directions of material deformation. Very small values of plastic deformation determined by means of numerical simulations, in which the material is treated as a solid body, may not even be detectable experimentally. Based on Figure 16c, it can be concluded that only the layer in which the material deformation is greater than approximately ε = 0.074 (Figure 15) and ε = 0.022 (Figure 17) can be detected by measuring the deformation of the ring profile (Figure 9b).

**Figure 18:**
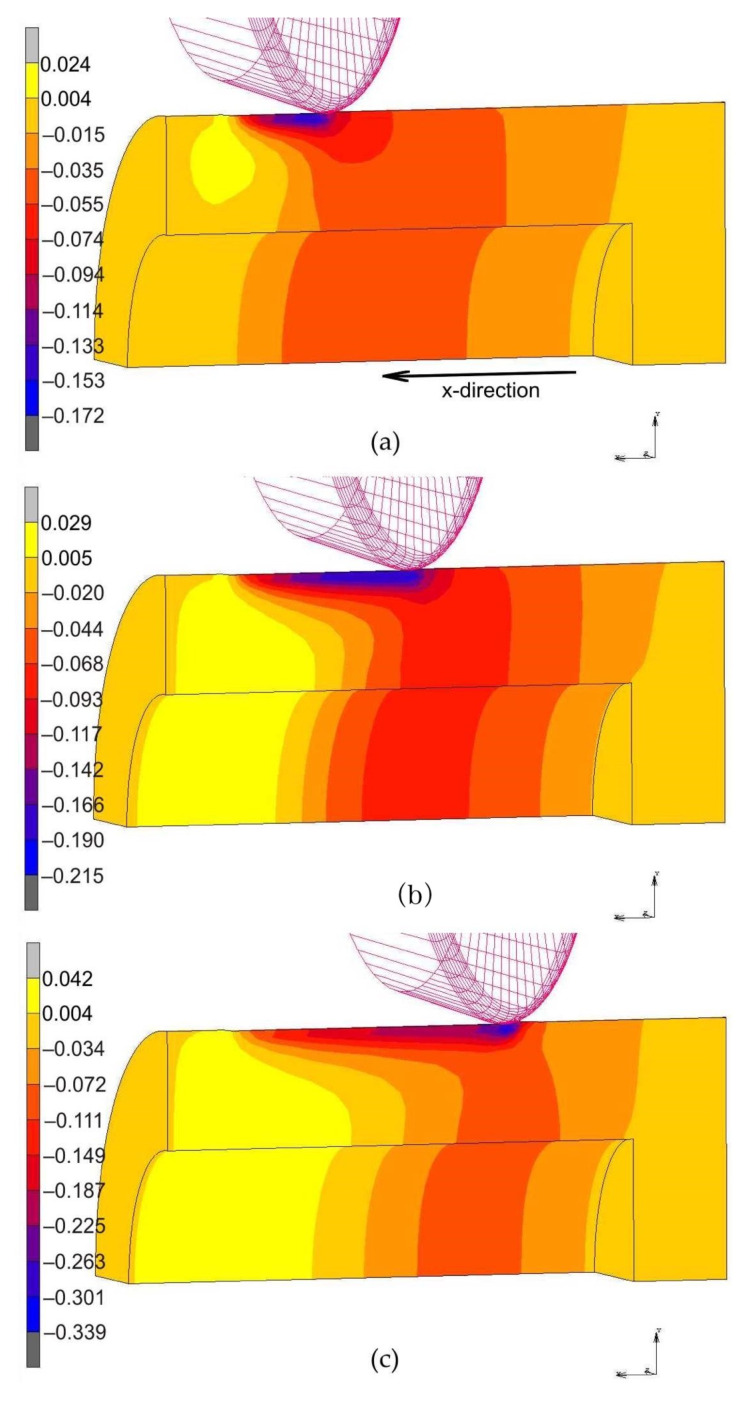
Displacement of the material in the x-direction during roller burnishing with braking moment across the width of the rings packet at a distance of: (**a**) 10 mm, (**b**) 20 mm and (**c**) 30 mm.

**Figure 19:**
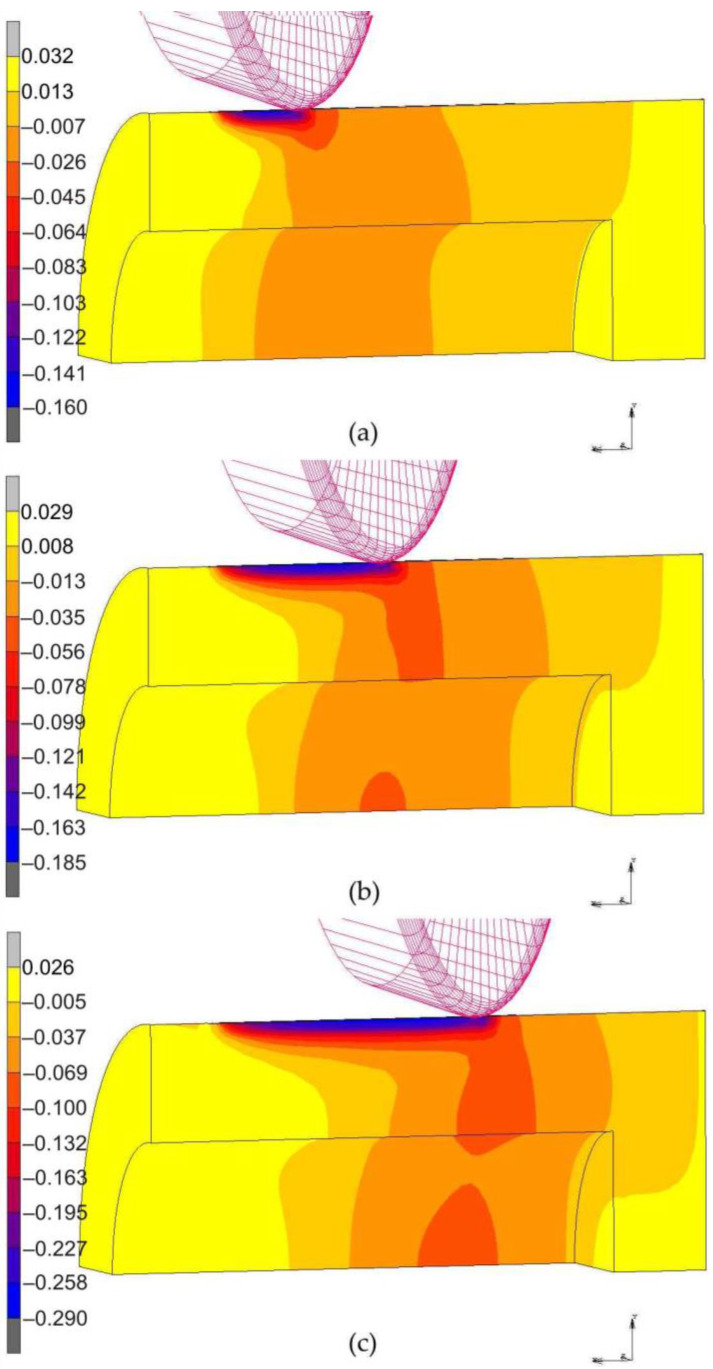
Displacement of the material in the x-direction during roller burnishing without braking moment across the width of the rings packet at a distance of: (**a**) 10 mm, (**b**) 20 mm and (**c**) 30 mm.

Figure 18 presents displacement of the material in the x-direction along the mandrel axis. The results of the displacement of the burnished material in the surface layer in the direction of the burnishing confirm the effectiveness of the proposed method of measuring the deformation of the rings to determine the thickness of the plastically deformed layer. The greatest deformations of the material are just accumulated under the roller and at the end of the rolling process they even reach an absolute value of 0.4 mm (Figure 18c). This leads to the formation of a distinct bulge of workpiece material in front of the roller which moves with the roller. Despite the fact that the distribution of material displacements suggests the displacement of the material along the entire rolled cross-section, most of them are elastic strains, which is confirmed by the distribution of places of occurrence of equivalent plastic strains (Figure 18 and Figure 19). In the case of roller burnishing without braking moment (Figure 19), the material flow is more uniform across the width of the set of rings than for roller burnishing with braking moment (Figure 18).

**Figure 20:**
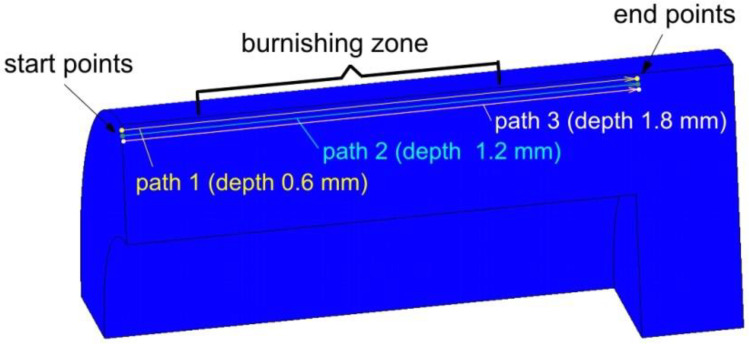
Scheme for measuring the distribution of equivalent plastic strains in numerical model.

**Figure 21:**
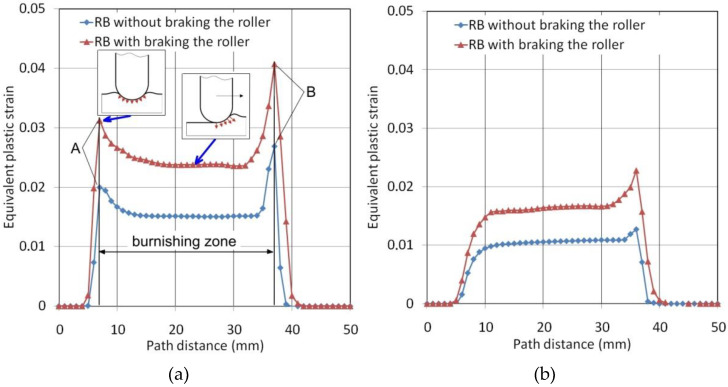

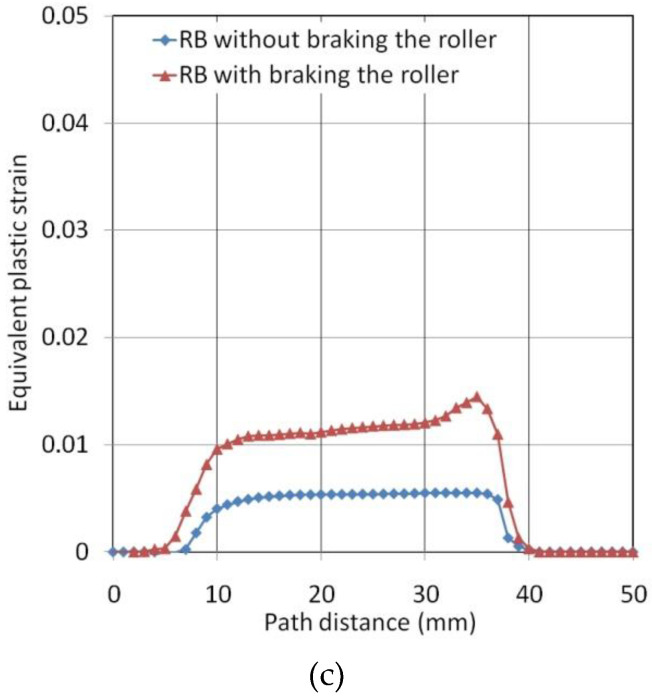
Distribution of the equivalent plastic strains along paths located at a depth of (**a**) 0.6 mm, (**b**) 1.2 mm and (**c**) 1.8 mm.

In order to compare the effect of applying the braking torque to the roller, we carried out a comparison of the distribution of equivalent plastic strain along the paths located at three different depths 0.6, 1.2 and 1.8 mm (Figure 20). In the initial stage of indenting the roller into the ring surface, a local increase in the value of equivalent plastic strain (point A in Figure 21a) was observed in the workpiece layer at a depth of 0.6 mm. This is because the roller contacts the workpiece surface with its entire surface during the indentation step. At this stage conditions for the formation of large plastic deformations occur directly below the surface of the roller. After starting the burnishing process by axial displacement of the roller, the contact surface of the roller with the rings is reduced by about half, which causes a further reduction in the value of equivalent plastic strains and their stabilisation. The character of changes in equivalent plastic strains is similar for both RB variants with and without braking the roller. However, the strains during RB with braking the roller achieve greater values across the entire area of the burnishing zone. Movement of the roller in relation to the material, which is a combination of burnishing speed and feed motion, causes most of the flowing material to accumulate in front of the tool. The creation of an additional material structure in front of the tool is noted in the literature and in workshop slang is referred to as the “jumping wave” [36,37] or “the wave of plastically deformed material” [22]. The equivalent plastic strain values in the zone of the “jumping wave” have much higher values (point B in Figure 21a) than in the stable burnishing zone. Due to the accumulation of the material in front of the roller, plastic deformations take place about 2 mm in front of it. The increased values of plastic strain at the stage of indenting the roller into the workpiece were obliterated at a layer depth of 1.2 mm (Figure 21b). The intense plastic deformation of the material immediately in front of the roller is still visible to some extent at this depth. It is obvious that the deformation values decrease with increasing depth of measurement (Figure 21c).

The aim of the research was to increase the depth of the plastically deformed layer and at the same time to obtain a surface without defects in the form of microcracks or micro pits. The application of the braking torque allowed a greater depth of the plastically deformed layer to be obtained with the same pressure force when compared to roller burnishing without braking the roller. Burnishing with braking takes place in the asymmetrical compression state of stress, which makes the material easier to transform into a plastic state.

## 4. Conclusions

Based on the experimental tests conducted and numerical simulations of the strengthening of the surface layer of the shafts using the new method of roller burnishing with the braking torque of the roller, the following conclusions can be drawn:

- The main problem in determining the depth of the plastically deformed layer is the difficulty of separating the plastic and elastic deformation zones. The plastically deformed layer can be divided into two characteristic areas: (i) the surface layer, which is characterised by a large deformation of the grains in the metallographic structure and (ii) a subsurface area of 2–3 times greater hardness, and whose microstructure is clearly different from the base material.
- The subsurface layer is slightly harder than the base material, and the microscopic image shows slight deformation of the metallographic structure. Two-times greater depth of plastic deformation was obtained using the method developed by the authors, which involves observation of the sample ring faces.
- The results of the measurement of the depth of the plastically deformed layer on the ring faces were consistent with the results of FEM simulation. FEM results confirmed the possibility of determining the depth of plastic deformation during roller burnishing based on the measurement of the deformation of the ring faces.
- The depth of the plastically deformed layer during roller burnishing can be increased to approx. 20% by applying a braking moment to the burnishing roller. Burnishing with roller braking takes place in a state of asymmetric triaxial compression and the deformation of the material is more effective than when using the same pressure force in conventional roller burnishing.
- Determining the depth of plastic deformation in the surface layer in the burnishing process using a package of rings is a process suitable for use in industrial conditions because it does not require the use of complicated laboratory equipment.

## Figures and Tables

**Table 1:** Selected mechanical properties of the C45 steel used in the tests.

Parameter	Value
Yield stress *R_e_*	400 MPa
Ultimate tensile stress *R*_m_	690 MPa
Elongation *A*_5_	19%
Young’s modulus *E*	2.12 × 10^5^ MPa
Poisson’s ratio *ν*	0.3
Strength coefficient *K*	1086 MPa
Strain hardening exponent *n*	0.124
Hardness HV1	180

**Table 2:** Chemical composition of C45 steel (wt.%) [30].

C	Si	Mn	Cr	Ni	Mo	Cu	S	P
0.42–0.50	0.1–0.4	0.5–0.8	max. 0.3	max. 0.3	max. 0.1	max. 0.3	max. 0.04	max. 0.04

**Table 3:** Dimensions of samples and burnishing parameters.

Parameter	Value
Outer diameter of the ring *d*	70 mm
Inner diameter of the ring *d*_i_	30 mm
Thickness of ring *g*	18 mm
Diameter of burnishing roller *D*	100 mm
Rounding radius of the burnishing roller *r*	1.5 mm
Burnishing speed *v*	42 m/min (180 rpm)
Feed rate *f*	300 mm/min

**Table 4:** The results showing the depth of the plastically deformed layer δ for the various burnishing forces.

Burnishing Force *F*, kN	Rolling Resistance Force *R*, N	Depth *δ*, mm
1	3.5	0.35
2	5.6	0.63
3	8.7	0.86
4	20.6	1.12
5	56.2	1.24

**Table 5:** The results of the depth of the plastically deformed layer *δ* for the various burnishing forces (burnishing with braking torque).

Burnishing Force *F*, kN	Rolling Resistance Force *R*ʙ, N	Slide Burnishing Force, N	Depth *δ*, mm
1	8.6	15.3	0.46
2	31.3	54.7	0.86
3	72.4	89.5	1.12
4	91.5	126.3	1.42
5	118	132.5	1.64

## Data Availability

The data presented in this study are available on request from the corresponding author.
